# Familial hypercholesterolemia and COVID-19: A menacing but treatable vasculopathic condition

**DOI:** 10.1016/j.athplu.2021.08.001

**Published:** 2021-08-08

**Authors:** Alpo Vuorio, Timo E. Strandberg, Frederik Raal, Raul D. Santos, Petri T. Kovanen

**Affiliations:** a Mehiläinen, Airport Health Center, Vantaa, Finland; b University of Helsinki, Department of Forensic Medicine, Helsinki, Finland; c University of Helsinki and Helsinki University Hospital, Helsinki, Finland; d University of Oulu, Center for Life Course Health Research, Oulu, Finland; e Faculty of Health Sciences, University of Witwatersrand, Johannesburg, South Africa; f Lipid Clinic Heart Institute (Incor), University of São Paulo, Medical School Hospital, São Paulo, Brazil; g Academic Research Organization, Hospital Israelita Albert Einstein, São Paulo, Brazil; h Wihuri Research Institute, Helsinki, Finland

**Keywords:** Cholesterol, COVID-19, Diabetes, Endothelial dysfunction, Familial hypercholesterolemia, PCSK9 inhibitor, Statin

## Abstract

SARS-CoV-2 infection continues to cause increased morbidity and mortality, and due to the slow pace of vaccination COVID-19 will probably remain a global burden to health systems for a long time. Unfortunately, the necessary prevention and treatment strategies of COVID-19 have led to restriction measures that are hampering the routine care of common chronic metabolic conditions like hypercholesterolemia. It is of particular concern that during the acute phase of COVID-19, the control of pre-existing metabolic diseases tends to get worse which again increases the risk for complications and a poor outcome in these patients. A significant contributor to these complications is endothelial dysfunction which is associated with COVID-19. This Commentary will discuss the impact of COVID-19 on endothelial function particularly in patients with familial hypercholesterolemia (FH), a metabolic inherited disease known to in itself adversely affect endothelial function. There should be no hesitation to continue with statin therapy in severe hypercholesterolemic patients with COVID-19. We argue that in FH patients with COVID-19 the clinicians need even consider intensifying statin therapy as well as the addition of other lipid-lowering agents, such as proprotein convertase subtilisin/kexin type 9(PCSK9) inhibitors. In contrast to statins, the PCSK9 inhibitors lower lipoprotein(a) [Lp(a)] level, and, accordingly, these latter drugs need to be considered particularly in FH patients with an elevated level of Lp(a). This call applies to the in-hospital stay and also beyond. When considering that the vasculopathic effects of COVID-19 may persist, a long-term follow-up of individualized therapies in FH patients is warranted.

## Highlights

• During the acute phase of COVID-19, the control of pre-existing metabolic diseases tends to get worse.
• Familial hypercholesterolemia (FH) is the most common monogenic inherited metabolic disease.
• COVID-19 increases the risk of myocardial infarction in FH patients.
• In FH patients with COVID-19 the clinicians need to consider intensifying lipid lowering therapy.

## 1 The bidirectional connection between COVID-19 and metabolic diseases

The COVID-19 illness continues to cause increased morbidity and mortality, and due to the slow pace of vaccination COVID-19 will probably remain a global burden to health systems for a long time. Unfortunately, the necessary prevention and treatment strategies of COVID-19 have led to restriction measures that are hampering the routine care of common chronic metabolic conditions like hypercholesterolemia, hypertension, hyperglycemia (diabetes), and obesity [1]. It is of particular concern that during the acute phase of COVID-19, the control of pre-existing metabolic diseases tends to get worse which again increases the risk for complications and a poor outcome in these patients [[2], [3], [4]]. A significant contributor to these complications is endothelial dysfunction which is associated with COVID-19 [5]. Moreover, pre-existing metabolic diseases like obesity, diabetes, hypercholesterolemia, and hypertension are conditions that exert continuous stress on the endothelium. This Commentary will discuss the impact of COVID-19 on endothelial function particularly in patients with severe hypercholesterolemia, such as familial hypercholesterolemia (FH), a metabolic condition known to in itself adversely affect endothelial function. Short comments on other metabolic conditions associated with endothelial dysfunction are made.

Hospital mortality rates, and potentially also long-term mortality (“metabolic long COVID-19”) among COVID-19 patients are greater in those with known ASCVD. One of the fatal mechanisms is the widespread damage of endothelial cells due to direct viral infection combined with the high levels of circulating cytokines associated with COVID-19, which predispose to both macrovascular (venous and arterial) and microvascular thrombosis [6,7]. Particularly, the microvascular endothelium of vital organs is sensitive to combined infectious and immuno-inflammatory attacks which easily results in the activation of the coagulation cascade and occluding microthrombus formation and ultimately causes multiorgan dysfunction -- often with a fatal outcome. Indeed, generalized thrombotic microangiopathy and associated endothelial dysfunction typically develop in severe forms of the COVID-19 [8].

## 2 FH-patients with COVID-19 are at increased risk

Heterozygous familial hypercholesterolemia (HeFH) is the most common monogenic inherited metabolic disease. HeFH affects worldwide 1 of 330 individuals [9]. If left untreated, serum low-density lipoprotein cholesterol (LDL-C) concentrations in HeFH are about 2-fold higher from birth, and, therefore predisposing to premature ASCVD [10]. Therefore, for children with HeFH, statin therapy should ideally be initiated at 8–12 years of age [11]. In the more severe homozygous form of FH (HoFH), affecting approximately 1 of 300 000 individuals, LDL-C concentrations are increased 4- to 6-fold and often remain elevated despite drugs and LDL-apheresis [12]. Accordingly, these patients often have an onset of ASCVD in early childhood. Considering the prevalence of FH, it is estimated that among the >140 million COVID-19 patients so far reported worldwide, approximately 420 000 and 470 patients with HeFH and HoFH, respectively, have been infected. In patients with FH, the severe hypercholesterolemia, if left untreated, causes lifelong endothelial dysfunction and thereby likely renders the vascular endothelium, including both the epicardial and intramyocardial coronary arterial endothelium, particularly vulnerable to the infectious and immunoinflammatory attacks and ensuing thrombus formation inherent in COVID-19 [13]. This prediction is supported by recent results derived from a large US national database which show that COVID-19 increases the risk of myocardial infarction in FH patients with or without diagnosed ASCVD [14].

## 3 Cholesterol metabolism in COVID-19 – SREBP-2 at the crossroad

COVID-19 leads to acute host-dependent dysregulation and reprogramming of several key metabolic pathways, including those involving amino acids, glucose, and lipids. Such alterations, at the cellular or whole-body level, can be either pro- or antiviral [15,16]. Before the current pandemic, long-term alterations in cholesterol metabolism have been observed after SARS-CoV-1 infection [17]. Additionally, genetic variants of the LDL-receptor may further modulate cholesterol metabolism, as has been observed with hepatitis C infection [18]. Very recent data have shown that activation of the sterol regulatory element-binding protein 2 (SREBP-2), a master regulator of sterol and fatty acid synthesis, and which also activates proinflammatory cellular mechanisms, is related to the severity of the COVID-19−associated cytokine storm particularly in obese patients [19]. Interestingly, cultured fibroblasts from HoFH patients overexpress SREBP-2 [20]. Consequently, a COVID-19-triggered overexpression of SREBP-2 activity might further predispose FH patients to acute and long-term cardiovascular complications.

## 4 Familial hypercholesterolemia, lipoprotein(a), and diabetes – diverse mutual associations

The levels of Lp(a) are elevated more often in FH patients than in the general population, and, among such FH patients, the high levels associate with endothelial dysfunction from childhood [7,21,22]. Furthermore, as an LDL-like lipoprotein particle with proinflammatory and anti-fibrinolytic properties, Lp(a) may enhance the proinflammatory and prothrombotic effects associated with COVID-19 [23]. The *LPA* gene includes an interleukin-6 (IL-6) element and there is a positive correlation between IL-6 and Lp(a) levels [24,25]. Thus, SARS-CoV-2 infection may further increase an already elevated Lp(a) level. Since the high level of IL-6 acts as an important mediator of the cytokine storm, which adversely affects also endothelial function [26], a joint elevation of Lp(a) could act synergistically in increasing the endothelial dysfunction and associated increased risk of thrombus formation. Consequently, a COVID-19 patient with an elevated Lp(a) level, and especially those who also have FH, may be more prone to develop thrombotic complications. In addition, Lp(a) level may remain elevated for several months after viral infection and may also destabilize pre-existing atherosclerotic plaques [27,28]. Therefore, the patients may remain at an increased risk for acute CVD events after discharge from the hospital [29]. Furthermore, it has been shown in a case-control study that COVID-19 relates to an increased arterial pulse wave velocity which reflects an increase in the arterial stiffness [30]. The authors speculate that such an increase in the stiffness is a consequence of a damage of the arterial endothelium caused by the SARS-CoV-2-induced systemic hyperinflammatory cytokine release and a direct viral damage of the endothelium. These insults, again, will reduce the bioavailability of NO and lead to structural changes in the arterial wall, thereby impacting vascular homeostasis and the balance of vasoconstriction and vasodilatation. Ultimately such imbalance might be related to worsening of the prognosis of the COVID-19 patients [30].

Endothelial function is also compromised in patients with type 1 or type 2 diabetes [31]. Moreover, SARS-CoV-2 infection-triggered new-onset diabetes may further aggravate glucose-dependent endothelial dysfunction [32,33], and COVID-19 may unmask existing vulnerabilities of endothelial cells in patients with type 2 diabetes [34]. Accordingly, any adjunctive therapies conferring endothelial protection against the diverse damaging elements are of paramount importance when a patient has a metabolic condition associated with endothelial dysfunction.

To further study the challenging associations between COVID-19 and new-onset diabetes, an international group of diabetes researchers has initiated the CoviDIAB Project [2]. Of note, the prevalence of type 2 DM is reported to be reduced in FH, but the mechanisms have remained unresolved [35]. Statins may affect glucose metabolism and even increase the incidence of new-onset diabetes in the general population [36,37], but no such statin-dependent increased risk of new-onset diabetes has been reported in FH patients [38]. Thus, including statin-treated FH patients with COVID-19 in the CoviDIAB Project could offer an interesting clinical model when trying to understand the relationship between new-onset diabetes and COVID-19.

## 5 Impact of lipid-lowering medications on sustained metabolic alterations in FH patients with COVID-19

Albeit no data are available currently, we can infer that COVID-19 triggers, inaddition to an acute, also sustained increase in the risk of cardiovascular events especially in FH patients [13,39]. Due to the expected presence of subclinical ASCVD and the associated increased mortality risks among adult FH patients with COVID-19, treatment of hypercholesterolemia must be continued and probably even intensified, not only acutely, but also for an extended period after COVID-19 infection. The need for effective treatment applies especially to statins, which have been associated with a more favorable prognosis in most studies among hospitalized COVID-19 patients [40]. Indeed, most retrospective analyses about mortality in hospitalized patients with COVID-19 suggest that ongoing statin therapy improves the prognosis of hospitalized COVID-19 patients [41]. This may be due to the anti-inflammatory and antithrombotic effects associated with statin treatment [42], although the greatest benefit of statins probably derives from their long-term vasculoprotective effects. Based on current expert opinion, the need to continue cholesterol-lowering therapy during and after COVID-19 is widely accepted even in non-FH hypercholesterolemia and other forms of dyslipidemia [13,43].

## 6 COVID-19 vaccines and familial hypercholesterolemia

Myocarditis/pericarditis has been reported after an mRNA COVID-19 vaccination in very rare cases among males aged 12-51-years [[44], [45], [46]]. The U.S. Centers for Disease Control and Prevention has estimated that the rate of these complications is about 12.6 cases per million doses of the second dose of mRNA COVID-19 vaccination. Although the mechanism behind these complications is not clear, it has been speculated that they are due to an immune response to the genetically engineered mRNA in the vaccine, which then would further activate immunological pathways. However, so far there are no reports of FH patients with myocarditis after COVID-19 vaccination. In any case, the benefits of COVID-19 vaccinations clearly overweight the risk of rare adverse events related to the vaccination. We reason that this paradigm also applies to patients with FH.

In conclusion, there should be no hesitation to continue with statin therapy in severe hypercholesterolemic patients with COVID-19, whether they are FH patients or not. We argue that in FH patients with COVID-19 the clinicians need even consider intensifying statin therapy as well as the addition of other lipid-lowering agents, such as ezetimibe and PCSK9 inhibitors. On top statins, the PCSK9 inhibitors lower serum Lp(a) level by about 30% and serum LDL-C by about 60% [43]. Thus, PSCK9 inhibitors need to be considered particularly in FH patients with an elevated level of Lp(a) [43]. In addition, PCSK9 inhibitors may potentially enhance the antiviral function of endogenously produced interferon [47].

This call for an effective hypolipidemic prevention applies during the time of the COVID-19 pandemic and beyond. The length of the vulnerable period requiring intensification of LDL-cholesterol-lowering therapy cannot be predicted at the moment. However, we need to recognize that cardiovascular diseases and FH are among the comorbidities that carry a high risk of complications for COVID-19 patients [14,48]. When considering that the vasculopathic effects of COVID-19 may persist, a long-term follow-up of individualized therapies in FH patients is warranted. It would be also important to collect epidemiologic follow-up data of the FH patients who have suffered COVID-19, and then analyze the benefits of a long-lasting particularly effective lipid lowering therapy [49,50].

## Funding

Dr. Santos is a recipient of a scholarship from the Conselho Nacional de Pesquisa e Desenvolvimento Tecnologico (CNPq) process # 303734/2018-3, Brazil.

## Author contributions

AV and PTK wrote the first draft of the manuscript. All authors listed have made a substantial, direct, and intellectual contribution to the work, and approved it for publication.

## Declaration of competing interest

Dr. Vuorio reports no conflicts of interest.

Dr. Kovanen has received consultancy fees, lecture honoraria, and/or travel fees from Amgen, Novartis, Raisio Group, and Sanofi.

Dr. Strandberg has had educational, consultative, and research collaborations with several companies (incl. Amgen, Merck, OrionPharma, Sankyo, Servier, Sanofi) marketing cholesterol-lowering drugs.∖

Dr. Santos has received honoraria related to consulting, research, and or speaker activities from Abbott, Amgen, Aché, Astra Zeneca, EMS Esperion, GETZ Pharma, Hypera, Kowa, Libbs, Merck, MSD, Novo-Nordisk, Novartis, PTC Therapeutics, Pfizer, and Sanofi.

Dr.Raal has received research grants, honoraria, or consulting fees for professional input and/or lectures from Sanofi, Regeneron, Amgen, and Novartis.
